# Funoran as a marine anti-biofilm polysaccharide for caries prevention: biological basis, current evidence, and translational challenges

**DOI:** 10.3389/fmicb.2026.1859856

**Published:** 2026-06-19

**Authors:** Fajian Guo, Lu Lu, Linhong Song, Yuge Jiao, Degang Sun

**Affiliations:** 1 Qingdao Stomatological Hospital Affiliated to Qingdao University, Qingdao, Shandong, China; 2 Qingdao Central Hospital, University of Health and Rehabilitation Sciences (Qingdao Central Hospital), Qingdao, Shandong, China

**Keywords:** biofilm inhibition, caries prevention, dental caries, funoran, marine polysaccharides, natural products, oral biofilm, *Streptococcus mutans*

## Abstract

Dental caries is a chronic biofilm-mediated disease that is caused by the interplay of plaque dysbiosis, persistent acid generation by cariogenic bacteria and host-associated environmental influences. Accordingly, current anti-caries measures increasingly focus on broader interventions targeting bacterial adhesion and colonization, biofilm formation, extracellular polysaccharide (EPS) production, and the local oral microecological equilibrium. Funoran, a sulfated polysaccharide derived from red algae, has become a promising natural marine bioactive, because it has desirable biocompatibility, interfacial activity, and potential anti-adhesive and antibiofilm properties. Current evidence suggests that funoran can reduce the adhesion of oral streptococci to tooth-relevant surfaces and may improve biofilm inhibition when combined with agents such as xylitol. Studies on structurally related algal polysaccharides further support the potential of marine polysaccharides in antibacterial, antibiofilm, anti-inflammatory, and oral delivery applications. Nevertheless, the development of funoran for caries prevention is still constrained by limited direct evidence, unclear mechanisms, non-standardized evaluation systems, and insufficient clinical research. Future studies should emphasize structural characterization, mechanistic investigation, standardized biological evaluation, and local delivery design to accelerate its translation into precision caries prevention.

## 1 Introduction

Dental caries is one of the most common chronic oral diseases worldwide. Rather than being a localized infection caused by a single pathogen, it is now widely recognized as a process of oral microecological dysbiosis resulting from the long-term interaction among dental plaque biofilms, fermentable sugar intake, host factors, and time (Lemos et al., 2019; Simon-Soro and Mira, 2015). Although streptococci, particularly *Streptococcus mutans*, were long regarded as the principal etiological agents of caries (Banas and Drake, 2018; Mattos-Graner et al., 2014), accumulating evidence suggests that caries should be more appropriately understood as an ecological disease driven by multispecies interactions, biofilm maturation, and the persistent maintenance of an acidic microenvironment (Spatafora et al., 2024; Zeng et al., 2026; Zhang et al., 2022). In this process, *S. mutans* and *Streptococcus sobrinus* play central roles in adhesion, extracellular polysaccharide (EPS) synthesis, and acidogenicity (Fragkou et al., 2016; Metwalli et al., 2013), while *Actinomyces viscosus* and *Lactobacillus acidophilus* are also involved in the progression of cariogenic demineralization (Matias Regis et al., 2025; Wei et al., 2024). In addition, associated microorganisms such as *Candida albicans* and *Veillonella parvula* may further enhance cariogenicity through synergistic interactions, biofilm stabilization, and acid tolerance adaptation (Koo and Bowen, 2014; Lu et al., 2023).

This updated understanding of caries pathogenesis has also reshaped the conceptual strategy of anti-caries intervention. Instead of concentrating on the growth inhibition of bacteria, recent approaches increasingly consider comprehensive interference with major cariogenic biofilm processes. The traditional preventive measures, such as fluorides, chlorhexidine, sugar substitutes, probiotics, and regular mechanical cleaning, have demonstrated apparent clinical usefulness. Nevertheless, they continue to have important Limitationss regarding long-term safety, local targeting, sustained activity in complex biofilms, and maintenance of the oral microecological balance. On this basis, natural products have become a significant trend in anti-caries research (Cheng et al., 2015; Jeon et al., 2011). Specifically, bioactive compounds of marine origin are gaining increasing popularity because their beneficial effects may go beyond antimicrobial proliferation control to anti-adhesion, anti-EPS formation, anti-acidogenicity, and anti-virulence control capabilities (Chen et al., 2020).

Red algae and their sulfated polysaccharides have been of long-standing interest within marine natural products due to their good biocompatibility and interfacial activity (Cotas et al., 2020; Perez et al., 2016), their film-forming properties and possible antibacterial and anti-inflammatory activities (Aziz et al., 2021; Huang et al., 2021; Lomartire and Goncalves, 2022; Matin et al., 2024; Wang et al., 2026). Of particular interest in this regard is funoran, a representative high-molecular-weight sulfated polysaccharide derived from red algae of the genus Gloiopeltis. Current literature indicates that funoran has the ability to prevent oral streptococcal adhesion on saliva-coated surfaces of hydroxyapatite (Inagaki et al., 2011), and that it may be more effective as an antiplaque agent when combined with xylitol (Nakamura et al., 2022; Wang et al., 2014). Beyond direct bioactivity alone, funoran may have value beyond that of conventional low-molecular-weight natural products; in addition to serving as a bioactive molecule, it may be used in material design and local delivery, giving it dual research importance in both bioactivity and oral materials.

The existing evidence remains fragmented and is mostly confined to *in vitro* observations, short-term application studies, or indirect evidence based on related algal polysaccharides. Consequently, there is no consistent analytical approach that can be used to relate cariogenic processes, direct evidence, and translational implementation. This points to an important scientific gap: funoran should not simply be tested as a natural antibacterial agent, but rather analyzed in the context of the pathological process of cariogenic biofilm formation and anti-caries treatment. Here, funoran serves as the focal point of the current article and within the wider context of caries etiology, cariogenic biofilm processes, developments in algal natural products and translational applications to oral materials, this study systematically reviews the *in vitro* data on its effects on key oral cariogenic microorganisms. It also discusses current research hotspots, existing Limitationss, and future prospects in this area. In this way, this review offers a more comprehensive view of the scientific value and translational potential of funoran in anti-caries studies.

## 2 Current research status

### 2.1 Basis of caries development and the roles of major cariogenic microorganisms

In this context, *S. mutans* is the most representative functional core microorganism due to its high tooth-surface adhesion, production of EPS dependent on the presence of sucrose, intense acidogenicity, and aciduricity, which facilitates its establishment and maintenance of a local microenvironment favorable to caries development (Lemos et al., 2019; Mattos-Graner et al., 2014). Recent data, however, show that the presence or colonization burden of *S. mutans* alone cannot explain the entire range of caries phenotypes (Mazurel et al., 2025). Other population and oral microbiome surveys have demonstrated that *S. sobrinus*, *A. viscosus*, and *L. acidophilus* could also play significant roles in cariogenesis and that their contribution to disease progression can differ depending on disease stage, age, and degree of lesion severity (Fragkou et al., 2016; Hoceini et al., 2016; Nagarathna and Veena, 2020). Moreover, non-mutans bacterial and fungal species have also been demonstrated to play an increasingly important role in the ecology of caries (AlEraky et al., 2021; Korona-Glowniak et al., 2022; Najafi et al., 2022; Zhang et al., 2025).

The synergistic association between *S. mutans* and *C. albicans* has been recognized as one such example because of its ability to increase the stability of biofilms, the formation of the EPS network, and the maintenance of a more devastating acidic microenvironment (Koo and Bowen, 2014; Lu et al., 2023). All these impacts are directly linked to the phenomenon of greater invasiveness of early childhood caries and severe carious lesions (Matias Regis et al., 2025). Similarly, other related bacteria such as *V. parvula* cannot be considered as passive co-inhabitants since they can also extend biofilm virulence and cariogenic potential when interacting with mutans streptococci. Collectively, these results suggest that dental caries is not a unipathogenic process, but rather a complex dysbiotic process that is determined by predominant microorganisms, synergistic taxa, host factors, and dietary exposure (Wei et al., 2024).

The implications of this etiological change on the testing of funoran as a possible anti-caries agent are important. If future research is confined to demonstrating that funoran inhibits the *in vitro* growth of a single *S. mutans* strain, it will be limited in terms of its applicability as an anti-caries agent since such results do not adequately indicate its applicability to the real cariogenic environment. By comparison, funoran would have much higher scientific and translational potential if it can suppress early adhesion, disrupt mature biofilm organization, inhibit multispecies coaggregation, or modulate the formation and maintenance of a localized acidic microenvironment. Thus, funoran cannot be considered as a typical natural antibacterial agent, but rather it must be evaluated in the context of caries initiation and progression, and it is important to consider the stages, targets, and the levels at which it can be effective. Overall, it is only through a re-assessment of funoran in the ecological paradigm of caries that the real potential of funoran in anti-caries studies and local oral care intervention can be precisely characterized.

### 2.2 Cariogenic biofilms and virulence mechanisms

Cariogenic biofilms do not simply arise as a by-product of bacterial aggregations, but are highly structured and dynamic systems that are structurally stable, metabolically cooperative, and functionally adaptive to the local microenvironment. One of the key events in the process is the cell wall-bound forms of glucosyltransferases secreted by *S. mutans* that synthesize EPS, which is dependent on the presence of sucrose and is critical to the formation of biofilm architecture as well as to the maintenance of its pathogenicity (Bowen and Koo, 2011). The resultant EPS matrix enhances bacterial adhesion and aggregation of bacterial cells on the tooth surfaces and, at the same time, changes the diffusion of the biofilm, thereby reducing the diffusion of buffering agents, and facilitating the retention of acidic metabolites. These changes enable localized demineralization and increase the cariogenic potential of the biofilm. The pathogenicity of *S. mutans* can therefore not be ascribed to one virulence determinant, but instead reflects the interaction of surface adhesins, the glucosyltransferase system, acidogenicity, aciduricity and stress adaptation systems (Krzysciac et al., 2014). Biofilm formation and virulence expression should be considered closely intertwined parts of the same pathogenic process.

Natural bioactive substances have been tested against several interrelated functions of *S. mutans*, including cell proliferation, adhesion, EPS-enriched biofilm formation, acid production, and virulence-gene expression (Chen et al., 2016; Rudin et al., 2023). These studies indicate that candidate anti-caries agents should not be assessed only by their short-term antibacterial potency. Their value also depends on whether they can disrupt the progression from initial adhesion to biofilm maturation and subsequent virulence enhancement. Studies on EPS metabolism and glucosyltransferase-targeted strategies have strengthened this functional view of cariogenic biofilms (Lin et al., 2021; Zhang et al., 2021). Therefore, anti-caries interventions need to be evaluated by asking whether they weaken the structural foundation and virulence profile of the biofilm, rather than relying solely on conventional antibacterial indices.

Cariogenic biofilm development is also shaped by ecological interactions among multiple species and strains. In *S. mutans*, surface-associated proteins participate in biofilm maturation, while competitive and cooperative relationships among oral streptococci and probiotic organisms can alter spatial organization, colonization behavior, and community stability (Matsumoto-Nakano, 2018; Wasfi et al., 2018). These findings place *S. mutans*-centered models within a wider microbial-ecological context. Biofilm architecture and treatment response may vary with strain composition, substratum properties, and synbiotic exposure, suggesting that microecological heterogeneity contributes directly to cariogenic potential (Momeni et al., 2024; Dang et al., 2024). Evidence from mono- versus multispecies assays and arginine-based synbiotic models further shows that experimental context can change the apparent anticariogenic activity of a given intervention (Huang et al., 2025; Pudipeddi et al., 2025). Evaluation of funoran should extend beyond single-species *in vitro* systems. Where feasible, multispecies and multistrain coculture models would better approximate the oral ecological environment and improve the translational relevance of the results.

Therefore, for in vitro studies of funoran to be mechanistically informative, they should prioritize inhibition of adhesion, disruption of biofilm formation, reduction of EPS synthesis, and modulation of virulence-related traits as core endpoints, rather than relying solely on minimum inhibitory concentration (MIC) or minimum bactericidal concentration (MBC) measurements. In this context, a function-oriented evaluation model is more likely to capture the true anti-caries potential of funoran.

### 2.3 Research progress in red algae/algae-derived natural products and marine polysaccharides

One major Limitations of current research is that many investigations still focus primarily on nonspecific antibacterial activity or single-enzyme inhibition, without adequately linking these effects to the pathological processes underlying caries initiation and progression (Cheng et al., 2015; Jeon et al., 2011). More recent perspectives therefore argue that the value of natural products in caries prevention lies not in simply substituting for conventional broad-spectrum antimicrobials, but in providing multitarget interventions with lower ecological disturbance, which is more consistent with the contemporary view of caries as a dysbiosis-driven disease (Chen et al., 2020). Taken together, these considerations indicate that the evaluation of natural anti-caries agents should be aligned more closely with cariogenic mechanisms and translational applicability.

Within this evolving system, algal natural products have drawn increasing interest, especially compounds obtained from red algae. Their appeal lies not only in biological activity, but also in their usefulness as functional materials. The reported activities are diverse and do not fit neatly into a single antimicrobial category. They include membrane perturbation, inhibition of biofilm development, regulation of interfacial interactions, antioxidant effects, and wider health-related applications (Perez et al., 2016; Cotas et al., 2020; Matin et al., 2024). This combination of functions helps explain why red-algal products are being considered for functional foods, oral-care formulations, and locally applied biomaterials. On this basis, red algae can be viewed as a relevant and promising reservoir of anti-caries bioactive substances.

Within algal-derived compounds, polysaccharides occupy a particularly important position. Their sulfation pattern, molecular conformation, and physicochemical behavior can connect biological effects with gel- or film-forming properties (Liu et al., 2019; Pei et al., 2021). This feature gives them practical value in oral films, hydrogels, and nanocomposite delivery systems, where local residence time and controlled exposure of active components are central design considerations (Lomartire and Goncalves, 2022; Wang et al., 2026). In contrast to many small-molecule marine natural products, algal polysaccharides therefore provide a formulation-oriented platform that may combine biofilm modulation with material design.

Funoran is better understood through functional comparison with other marine polysaccharides than through direct equivalence to them. Carrageenan and porphyran offer red-algal reference points for sulfation-related activity and gelation. Fucoidan, by contrast, provides a brown-algal comparator supported by a broader translational literature covering inflammation, immune regulation, microbiome modulation, and recent human-use studies (Qiu et al., 2021; Garcia et al., 2025; Wang et al., 2024). Clinical work on carrageenan also indicates that sulfated marine polysaccharides can be feasible for mucosal use, although such evidence should not be taken as direct support for the anti-caries efficacy of funoran (Unger-Manhart et al., 2024). These comparisons are useful mainly because they frame funoran’s possible role while keeping the evidentiary boundary clear.

For oral-health applications, the direct evidence on funoran remains more limited. Available studies chiefly point to reduced adhesion of oral streptococci and enhanced biofilm suppression when funoran is used with xylitol (Inagaki et al., 2011; Nakamura et al., 2022). Its value should therefore not be presented as broader or better established than that of more extensively studied polysaccharides. A more cautious and defensible interpretation is that funoran may be distinguished by its anti-adhesive activity, compatibility with oral-care formulation needs, and potential dual role as both a bioactive polysaccharide and a local delivery material (Yang et al., 2022).

To clarify the relative position of funoran, Table 1 summarizes its source, key properties, main oral-health relevance, and evidence boundary alongside three related marine polysaccharides: carrageenan, porphyran, and fucoidan. This comparison places funoran within the broader context of sulfated marine polysaccharides while emphasizing that its current support is mainly linked to anti-adhesive activity and potential oral-care formulation use.

**Table 1:** Comparative positioning of funoran and representative natural anti-caries agents.

Agent	Main mechanism	Effective form/concentration	Safety/formulation
Funoran	Anti-adhesion; biofilm reduction with xylitol	Funoran-xylitol gum/tablets; independent dose range unclear	Likely formulation-friendly; oral safety data limited
Chitosan	Membrane/surface disruption; reduced *S. mutans* burden	Mainly chewing gum, rinse, particles, coatings	Biocompatible; activity depends on molecular weight (MW), deacetylation, pH
Alginate-based materials	Delivery matrix; possible biofilm/EPS interference	Mainly hydrogel, scaffold, composite systems	Low toxicity; strong material utility
Green tea catechins/EGCG	Reduced acid production, glucosyltransferase (GTF) activity, EPS, biofilm	Variable EGCG ranges; mouthrinse/in vitro systems	Taste, staining, stability, dose safety need control
Cranberry proanthocyanidins (PACs)	Anti-adhesion; reduced GTF/EPS and virulence	Extract/PAC fractions; reported in vitro mg/mL ranges	Sugar-free, low-acid formulation needed

Beyond comparisons within marine sulfated polysaccharides, funoran also needs to be placed alongside natural agents that have been examined more extensively in caries research. Chitosan is mainly discussed in relation to bacterial-surface disruption and reduction of salivary *S. mutans* levels (Róna et al., 2023). Alginate-based systems, by contrast, are more often considered for material design, local delivery, and polysaccharide-based dental applications (Yang et al., 2022; Lin et al., 2022). Green tea catechins provide another benchmark because epigallocatechin gallate (EGCG) has been linked to reduced *S. mutans* growth, biofilm formation, acidogenicity, and virulence-related activity (Aragão et al., 2022; Kong et al., 2022). Cranberry-derived proanthocyanidins have also been associated with anti-adhesion effects and interference with EPS or glucan-related biofilm development (Castellanos et al., 2024). Viewed against this literature, funoran is best positioned as an anti-adhesive, formulation-oriented polysaccharide. Its dose–response profile, oral-formulation safety, and clinical support, however, remain less mature than those of several better-characterized natural agents.

### 2.4 Putative mechanisms of funoran in anti-adhesion and EPS-matrix inhibition

Although the precise mode of action of funoran has not been fully resolved, the available evidence allows several mechanistic possibilities to be proposed with appropriate caution. The most direct hypothesis concerns early bacterial adhesion. Funoran is a high-molecular-weight sulfated polysaccharide with a polyanionic and hydrophilic backbone. These properties may enable it to form a hydrated interfacial layer on saliva-coated hydroxyapatite or on bacterial surfaces. Once such a layer is present, close contact between oral streptococci and the acquired pellicle may become less stable. Adhesion-related binding sites could also be partially shielded, and electrostatic or protein-mediated interactions required for initial attachment may be weakened. This interpretation is consistent with studies showing that funoran-containing formulations reduce the adherence of oral streptococci to saliva-coated hydroxyapatite (Inagaki et al., 2011). Evidence from other marine sulfated polysaccharides also supports the broader plausibility of antibacterial and antibiofilm activity against dental plaque-associated bacteria, although these findings should not be treated as direct proof of the same mechanism for funoran (Jun et al., 2018). The reported enhancement of biofilm inhibition when funoran is combined with xylitol further suggests that its functional value may be strongest in formulation-based systems rather than as a conventional bactericidal agent (Nakamura et al., 2022).

A second possible mechanism involves the EPS-rich biofilm matrix. In cariogenic biofilms, *S. mutans* glucosyltransferases synthesize water-soluble and water-insoluble glucans, which form the structural basis for bacterial accumulation, acid retention, and biofilm maturation (Lin et al., 2021). Interference with glucosyltransferase activity or glucan organization is therefore an important strategy for controlling *S. mutans* biofilm development (Zhang et al., 2021). Funoran may reduce EPS accumulation indirectly by limiting the number of bacterial cells that successfully attach during the early stage. This would decrease the local density of glucosyltransferase-producing cells and may subsequently weaken matrix formation. Another possibility is that funoran affects matrix assembly more directly. By analogy with related sulfated polysaccharides, it may interact with extracellular proteins, glucans, or divalent cations through charge-based binding. Such interactions could alter the hydration, diffusion properties, and spatial organization of the developing EPS matrix. At present, these mechanisms remain hypothetical for funoran. They should be tested by measuring water-soluble and water-insoluble EPS, glucosyltransferase activity, terminal biofilm pH, and the expression of genes related to adhesion, EPS synthesis, acid tolerance, and biofilm maturation.

The structure–activity relationship of funoran is also likely to be shaped by molecular weight, degree of sulfation, and spatial conformation. For sulfated polysaccharides, sulfate content, molecular-weight distribution, monosaccharide composition, and conformational features can all influence biological activity and material behavior (Cotas et al., 2020). A relatively high molecular weight may favor surface coating, steric hindrance, and local retention on tooth-relevant surfaces. Lower-molecular-weight fractions, by contrast, may diffuse more readily into early biofilm matrices. Degree of sulfation is another key variable. Increased sulfation may strengthen polyanionic interactions with bacterial surfaces, salivary proteins, and extracellular matrix components, but this relationship is unlikely to be simply linear. Excessive charge density or altered conformation may also affect solubility, viscosity, and molecular accessibility. Structural studies of red-algal sulfated polysaccharides support the need to evaluate these parameters together rather than separately (Pei et al., 2021). Recent work on marine polysaccharides similarly emphasizes that biological function often reflects the combined effects of molecular structure, conformation, and application context (Wang et al., 2026). Future research should compare funoran fractions with defined molecular-weight ranges, sulfate contents, and conformational characteristics. Such comparisons would help clarify whether its anti-adhesive and EPS-modulating effects mainly arise from interfacial shielding, direct matrix interference, or downstream regulation of cariogenic virulence.

### 2.5 Direct evidence for the effects of funoran and related algal polysaccharides on cariogenic microorganisms

**Figure 1:**
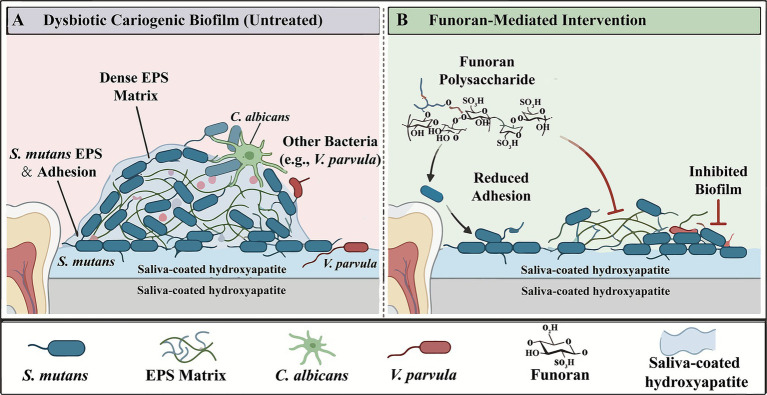
Schematic illustration of a dysbiotic cariogenic biofilm and the proposed intervention pattern of funoran. **(A)** In the untreated state, *S. mutans*-driven adhesion, EPS accumulation, and multispecies cooperation promote the development of a dense cariogenic biofilm on saliva-coated hydroxyapatite. **(B)** Funoran is proposed to reduce initial adhesion and suppress subsequent biofilm establishment, thereby weakening biofilm maturation and ecological pathogenicity.

Despite the limited number of direct studies on funoran, existing evidence has already provided preliminary support for its anti-caries potential. Xylitol chewing gum and tablets containing funoran have been shown to significantly reduce the adsorption of oral streptococci to saliva-coated hydroxyapatite, implying that funoran may function mainly by interfering with the initial stage of tooth-surface colonization rather than by acting as a conventional bactericidal agent (Inagaki et al., 2011). This mode of action is of particular relevance to caries prevention, as interference with early adhesion may reduce the colonization advantage and ecological competitiveness of cariogenic microorganisms while causing less disruption to the resident oral microbiota. Taken together, the currently available direct evidence suggests that anti-adhesion may represent one of the most relevant functional features of funoran in the context of caries control. As illustrated in Figure 1, the currently available evidence supports a model in which funoran primarily reduces early adhesion to saliva-coated hydroxyapatite and thereby attenuates the development of a dense EPS-rich cariogenic biofilm.

Available studies also support the feasibility of incorporating funoran into practical oral care formulations. Investigations of xylitol chewing gum containing funoran have shown effects on dental plaque, supporting its potential applicability in daily oral care products (Wang et al., 2014). Further evidence indicates that the combination of xylitol and funoran can inhibit *S. mutans* biofilm formation, implying that the advantages of funoran may be more evident in combination systems than in development as a single active component (Nakamura et al., 2022). On this basis, funoran can be provisionally regarded as a promising candidate for anti-adhesion and biofilm regulation. However, its direct effects on acidogenicity, EPS synthesis, virulence gene expression, and multispecies biofilm organization remain insufficiently characterized. Therefore, although the application prospects of funoran are increasingly plausible, its mechanistic basis still requires systematic validation.

Because the direct literature on funoran remains sparse, current assessments of its anti-caries potential necessarily rely in part on extrapolation from related algal polysaccharides. Systematic reviews have shown that marine algal sulfated polysaccharides possess antibacterial and antibiofilm activities against dental plaque-associated microorganisms (Jun et al., 2018). Related evidence further supports the broader capacity of algal sulfated polysaccharides to inhibit plaque bacteria and interfere with biofilm development (Achmad et al., 2020). In addition, marine algal bioactive substances have been reported to exert anti-caries-related effects directly against *S. mutans* (Sangavi et al., 2024). Although such findings cannot be considered direct proof of funoran activity, they do support the theoretical plausibility of a research strategy centered on marine polysaccharides for targeted intervention against cariogenic microorganisms. In this sense, the current rationale for funoran is constrained by limited direct evidence but strengthened by coherent indirect evidence.

Broader research on algal polysaccharides further supports the translational relevance of this field. Agar, carrageenan, and their composite systems have shown suitability for antibacterial films, functional composites, and delivery platform design, highlighting the material applicability of algal polysaccharides (Kanmani and Rhim, 2014; Shankar et al., 2015). Subsequent studies have extended this perspective to more advanced systems, including composite and functional material development (Kim et al., 2022; Madruga et al., 2020; Simona et al., 2021). Other work has shown that fucoidan-gold nanoparticles can inhibit biofilms formed by multiple microorganisms, further illustrating the potential of marine polysaccharides in biofilm control (Tabassum et al., 2023). From the perspective of oral implant materials, antibacterial polysaccharides have also been recognized as promising components in local material design (Hallmann and Gerngroß, 2025). At the same time, studies addressing virulence regulation and structure–function relationships caution that different algal polysaccharides, although partially comparable, should not be assumed to share identical mechanisms or equivalent effects (Palafox Felix et al., 2025; Wang et al., 2026). Accordingly, the most defensible conclusions regarding funoran currently remain focused on anti-adhesion and combination-based biofilm control, whereas high-quality evidence on MIC, MBC, minimum biofilm inhibitory concentration (MBIC), minimum biofilm eradication concentration (MBEC), virulence regulation, and validation in multispecies models is still lacking.

Overall, the current evidence base for funoran is characterized by limited direct support but relatively abundant indirect support. This pattern not only restricts the interpretation of existing findings, but also delineates the major directions for future research. Conclusions regarding funoran should clearly distinguish between effects directly demonstrated by existing studies and inferences derived from related algal polysaccharides. Research on algal polysaccharides has already established methodological pathways that can be directly adapted to funoran, including adhesion assays, mature biofilm models, composite-system development, and virulence-factor analysis. Therefore, funoran appears well positioned to progress toward a research stage in which mechanistic verification and translational development can be advanced in parallel. For clarity, the currently available direct evidence specifically related to funoran is summarized in Table 2.

**Table 2:** Summary of currently available direct evidence on funoran-related anti-caries effects.

Reference	Formulation/model	Target	Principal finding	Limitations
Inagaki et al. (2011)	Funoran-xylitol gum/tablets; saliva-coated hydroxyapatite model	Streptococcal adhesion	Decreased adhesion of oral streptococci	Adhesion-focused; limited mechanistic insight
Wang et al. (2014)	Funoran-xylitol chewing gum	Plaque-related outcome	Suggested plaque-reducing potential in oral-care formulations	Short-term and formulation-driven; limited microbiological detail
Nakamura et al. (2022)	Xylitol-funoran combination; *S. mutans* biofilm model	Biofilm formation	Reduced *S. mutans* biofilm formation	Single-species design; independent contribution of funoran unclear

### 2.6 Anti-caries applications, translational pathways, and methodological support

**Figure 2:**
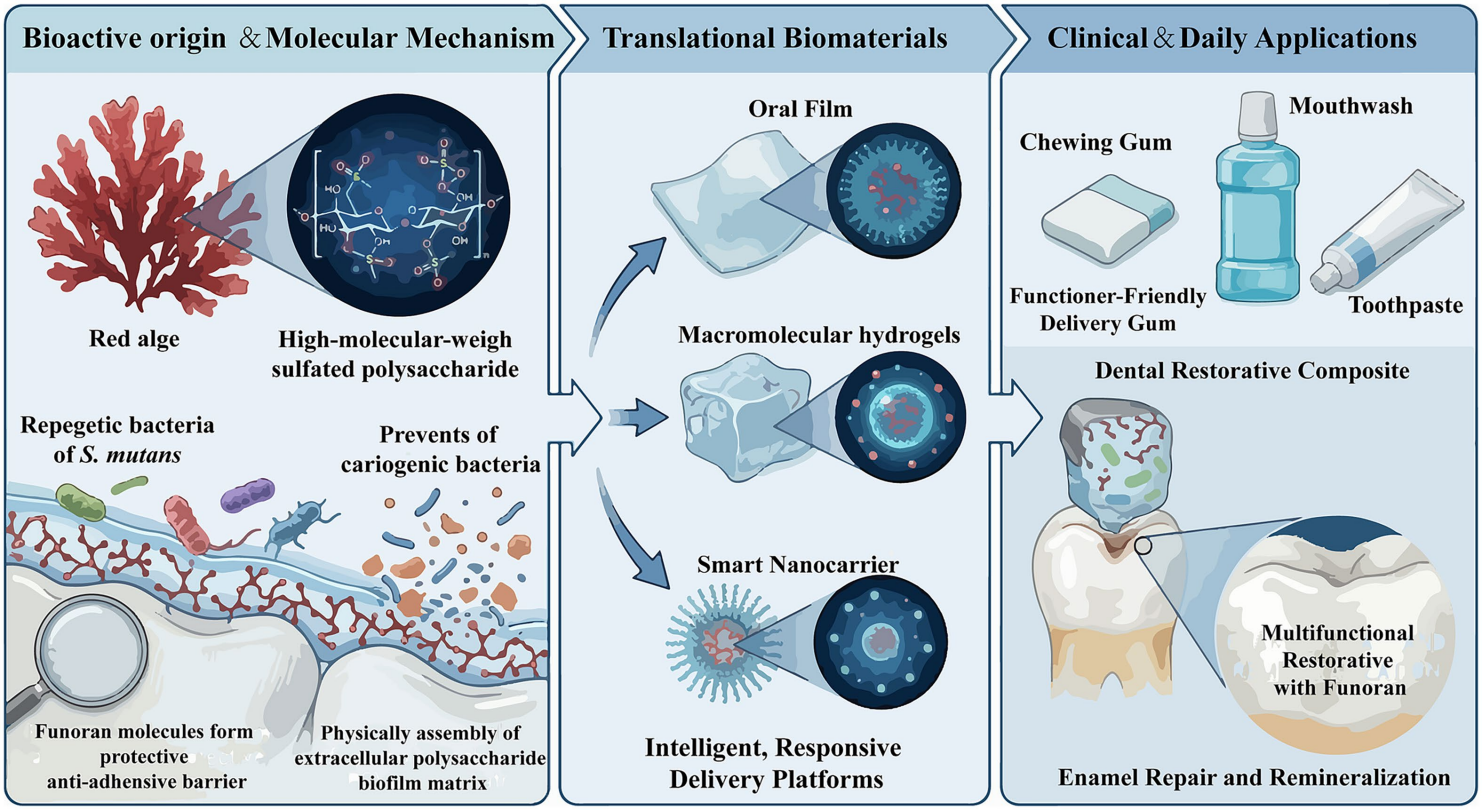
Conceptual overview of the bioactive origin, mechanistic basis, and translational application pathways of funoran. As a red algae-derived high molecular weight sulfated polysaccharide, funoran may contribute to anti-caries intervention through anti-adhesive and biofilm regulatory effects, while also supporting development of oral films, hydrogels, smart nanocarriers, daily oral-care products, and restorative composite systems.

The significance of funoran and related algal polysaccharides extends beyond *in vitro* activity screening to their potential development as local anti-caries formulations or functional oral materials. Chewing gum has been identified as an effective oral delivery vehicle because it prolongs the contact time between active agents and the tooth surface and is therefore suitable for low-dose, localized, and repeated administration (Wessel et al., 2016). Studies of funoran-containing xylitol chewing gum reflect this translational concept by using daily oral care carriers to enhance local retention and functional activity of bioactive components (Wang et al., 2014). Related evidence further indicates that the combination of funoran and xylitol can achieve biofilm-inhibitory effects in practical formulations (Nakamura et al., 2022). Nevertheless, the clinical translation of natural bioactive substances remains constrained by several barriers, including inadequate standardization of raw materials and formulations, limited strength of clinical evidence, and insufficient long-term safety validation (Ancuceanu et al., 2019). Overall, current evidence supports the practical applicability of funoran-based local intervention, while also underscoring the need to resolve key translational Limitationss. As summarized in Figure 2, the translational trajectory of funoran extends from its red algae-derived sulfated polysaccharide structure and anti-adhesive bioactivity to oral biomaterial design and daily anti-caries applications.

Recent advances in nanodelivery and intelligent responsive systems have created new technological opportunities for translating natural-product-based anti-caries strategies. Nanocatalyst-based approaches that promote degradation of the *S. mutans* biofilm matrix while enhancing bactericidal activity have been shown to improve intervention efficiency, highlighting the advantage of a “biofilm disruption followed by bacterial killing” strategy in cariogenic biofilm control (Gao et al., 2016). In parallel, the emergence of pH-sensitive polymeric micelles, *L. acidophilus* cell-envelope nanoparticles, and intelligent particles indicates that oral anti-caries formulations are shifting from passive release toward environmentally responsive and targeted controlled-release systems (Weng et al., 2022; Yi et al., 2020). This trend has been further reinforced by more recent evidence on intelligent particle systems for oral applications (He et al., 2024). Reviews have also emphasized the broad applicability of polysaccharides in oral delivery systems and pediatric dental materials (Katsarov et al., 2021; Yang et al., 2022). In this context, the macromolecular nature, hydrophilicity, and film-forming capacity of funoran suggest strong theoretical compatibility with these emerging platforms and support its potential transition from a bioactive compound to a functional material.

From a clinically oriented perspective, contemporary anti-caries strategies increasingly emphasize local microecological regulation rather than reliance on a single potent antibacterial effect. This shift is reflected in approaches based on synergy among multiple components, materials, and mechanisms (Lee and Kim, 2014; Perez-Diaz et al., 2015). Subsequent studies have further reinforced the importance of multi-target and multi-material strategies in caries management (Amissah et al., 2021; Zhang et al., 2020). In this context, funoran seems to have a number of feasible application routes. Possible uses of funoran consist of using it as an anti-adhesive or antiplaque ingredient in everyday oral care items, including chewing gum, mouthwash, toothpaste or sprays, to perform local intervention. Its polysaccharide backbone also allows incorporation into local delivery platforms, such as oral films, hydrogels, microspheres or nanocarriers, thus potentially improving oral retention and sustained release. In addition to application as a single active agent, funoran can be combined with xylitol, probiotics, metallic nanoparticles, or remineralizing agents to form composite systems with multiple capabilities in microbial control, biofilm control, and tissue repair. Thus, the translational potential of funoran does not simply consist of its own antibacterial activity, but also of its ability to be used as an active component of a system of local oral intervention in synergy.

Funoran may be incorporated into local oral delivery systems through several formulation routes that can be tested experimentally. Its film-forming polysaccharide structure makes it a plausible component of mucoadhesive oral films or dissolving strips. In such systems, funoran could provide anti-adhesive activity while also helping to form a matrix for xylitol, fluoride, calcium-phosphate remineralizing agents, or probiotics. Hydrogels offer a second route. A funoran-containing hydrogel could be designed for longer retention in pits, fissures, around orthodontic appliances, or at other plaque-retentive sites, thereby extending surface contact and supporting sustained anti-adhesion or pH-buffering combinations. A further possibility is to use funoran in nanocomposite platforms, such as polysaccharide-based nanoparticles, metallic nanoparticle composites, or remineralizing nanofillers, where surface-interference effects could be paired with controlled release or matrix-disruptive functions (Amissah et al., 2021; Chen et al., 2025; He et al., 2024). These examples should be viewed as formulation concepts rather than established clinical products, but they indicate how funoran research could progress from extract-level activity screening toward formulation-oriented anti-caries development.

The feasibility of such applications, nevertheless, will ultimately depend on methodological standardization and the quality of supporting evidence. MIC, MBC, biofilm determination, adhesion, and microscopic analyses are the most frequently used *in vitro* methods to measure the anti-caries effect of natural products (Galvao et al., 2012; Joycharat et al., 2012). These methods have also been extended to natural compounds and oral biofilm models in other studies, demonstrating the variety of available evaluation strategies (He et al., 2019; Niu et al., 2020). Recent studies still use similar endpoints, but significant methodological heterogeneity remains (Azarm et al., 2024; Song et al., 2020). Agar microdilution techniques, comparative antibacterial models, and the difference between MIC and MBEC all suggest that the lack of standardization prevents cross-study comparability (Brady et al., 2017; Golus et al., 2016; Schumacher et al., 2018). On this basis, funoran should not be assessed using a single endpoint. Further research is needed to compare both planktonic and biofilm conditions and include a more extensive list of indicators, such as MIC/MBC, MBIC/MBEC, bacterial adhesion, EPS production, acidogenicity, virulence gene expression, and microscopic structural changes. In conclusion, a multidimensional and standardized evaluation paradigm is needed to provide sufficient support for conclusions about the anti-caries value of funoran.

The oral environment is also complex, which further limits the translational relevance of static culture systems alone. Salivary flow, food residues, pH change, mechanical abrasion, and the dynamic turnover of multispecies communities may significantly affect the functionality of active substances on tooth and mucosal surfaces. This is why future research on funoran needs to gradually expand the use of artificial oral models, dynamic flow systems, three-dimensional biofilm models, and enamel or dentin-based substrates to test its anti-adhesive, antibiofilm, and acid-modulating properties under conditions that are more closely related to clinical reality. Moreover, batch variation, molecular-weight distribution, sulfation properties, experimental replication, and statistical analysis should be fully reported to enhance transparency, comparability, and trustworthiness of evidence synthesis. Hence, robust translational evaluation of funoran requires not only improved experimental models, but also more standardized methodological reporting and higher-quality evidence.

### 2.7 Standardized evaluation roadmap for future funoran studies

To improve comparability across future studies, funoran research should move from isolated antibacterial or biofilm assays toward a standardized evaluation roadmap. Material characterization needs to precede biological testing. Each study should report the algal source, extraction and purification procedure, molecular-weight distribution, degree of sulfation, monosaccharide composition, batch information, and solution properties, including viscosity and pH. These variables are not merely technical details, because differences in polysaccharide preparation may affect adhesion, diffusion, matrix interaction, and biofilm inhibition.

Early screening should combine planktonic assays with surface-associated models. MIC and MBC remain useful for defining baseline antibacterial activity, but they are insufficient for judging anti-caries potential. For funoran, saliva-coated hydroxyapatite and enamel- or dentin-based adhesion models deserve particular priority, since the strongest direct evidence relates to inhibition of oral streptococcal attachment. Key endpoints should include bacterial adhesion rate, viable cell counts, surface coverage, and early biofilm biomass. Experimental conditions such as bacterial strain, inoculum density, sucrose concentration, funoran concentration, exposure time, substratum type, and salivary pellicle preparation should be reported in sufficient detail to allow comparison across studies.

Mature biofilm models are needed to determine whether funoran affects established cariogenic biofilms and EPS-matrix development. Static microplate assays can serve as preliminary screens, but they should be complemented by three-dimensional (3D) biofilm models, flow-cell systems, or artificial oral models that better reflect salivary flow, nutrient exposure, and pH fluctuation. Priority readouts should include biofilm thickness, bacterial viability, live/dead distribution, total biomass, water-soluble and water-insoluble EPS, glucosyltransferase activity, acid production, terminal biofilm pH, and microscopic architecture. Whenever possible, confocal laser scanning microscopy (CLSM), crystal violet staining, colony-forming unit (CFU) counting, EPS quantification, and pH or lactic-acid assays should be combined, so that conclusions do not depend on a single endpoint.

Ecological relevance and reporting quality should be strengthened in parallel. Future studies should extend beyond single-species *S. mutans* models and include multispecies biofilms involving *S. sobrinus*, *Actinomyces* spp., *Lactobacillus* spp., *C. albicans*, or other cariogenic partners. *In situ* enamel-slab models, ex vivo substrates, and saliva-derived microcosm biofilms would be useful for testing whether funoran retains anti-adhesive or EPS-modulating effects under more clinically relevant conditions. These models should assess biofilm suppression together with microbial composition, acidogenicity, enamel demineralization, and compatibility with commensal oral microbiota. Unified reporting should include full dose–response curves, positive and negative controls, vehicle controls, replicate numbers, statistical methods, endpoint definitions, and clear separation of preventive treatment, co-treatment, and post-treatment designs. For formulation systems such as chewing gum, mouthwash, oral films, hydrogels, or nanoparticles, local retention, release profile, stability, cytocompatibility, taste acceptability, and enamel or mucosal safety should also be evaluated. This roadmap would make funoran studies more reproducible and help clarify whether it is best positioned as an anti-adhesive agent, an EPS-matrix modulator, or a component of local oral delivery systems.

## 3 Research hotspots and frontier trends

Cariology has been focused on the sustained attention to *S. mutans* biofilm formation, EPS metabolic networks, and glucosyltransferase-mediated regulation, which continue to be central to both mechanistic caries research and anti-caries strategy development (Lin et al., 2021; Zhang et al., 2021). More recent research also suggests that anti-caries interventions are no longer focused on simple growth inhibition, but are aimed at specific modulation of important processes related to virulence, such as adhesion, aggregation, matrix formation, and virulence expression (Momeni et al., 2024; Rudin et al., 2023). At the same time, greater attention has been given to interspecies interactions and their synergistic effects on pathogenicity, especially those among *S. mutans*, *C. albicans*, and *V. parvula*, which are closely linked to the virulence of severe caries and early childhood caries (Koo and Bowen, 2014; Lu et al., 2023; Matias Regis et al., 2025; Wei et al., 2024). For this reason, future funoran studies should be expanded to include multispecies systems to explore its impact on adhesion dynamics, biofilm architecture, and metabolic interactions in multispecies environments.

Previous studies mainly concentrated on extract-based screening and descriptive evaluation of biological activity, but more recent studies are increasingly concentrating on structure–activity relationships, composite system design, and material-oriented applications (Achmad et al., 2020; Jun et al., 2018). This shift implies that funoran may have research value not only because of its possible antibacterial or antibiofilm properties, but also because of its molecular structure, physicochemical properties, and functionality in multicomponent systems (Behzadnia et al., 2024; Wang et al., 2026). Thus, funoran should be considered a functional algal polysaccharide, rather than a marine-based antibacterial candidate discovered by activity screening alone.

Several developments in oral anti-caries technologies at the translational level further broaden the potential role of funoran as a bioactive substance to a component of a material and delivery. The use of nanodelivery platforms, intelligent particles, hydrogels, and polysaccharide-based composite materials has emerged as significant localized oral intervention delivery systems (Amissah et al., 2021; Weng et al., 2022). This trend indicates a broader expansion in the scope of natural polysaccharides, which are increasingly being used not only as active agents but also as structural materials or delivery-platform components (Chen et al., 2025; He et al., 2024). Since funoran is macromolecular, hydrophilic, and able to form a film, it has potential in local caries prevention not only due to its biological activity, but also due to its ability to modulate and release drugs or growth factors, sustain functional materials, and build nanopores. Meanwhile, current anti-caries studies are shifting away from elimination of cariogenic microorganisms toward control of the oral microecological balance. In line with such transformation, there is a growing trend in research on combined interventions, using sugar substitutes, probiotics, natural products, and polysaccharides, which focus on selective modulation instead of sterilization in general (Lee and Kim, 2014; Liang et al., 2024; Motallaei et al., 2021; Wasfi et al., 2018). These findings provide a broader translational template for determining the most suitable functional role of funoran in modern oral care systems.

Here, it is more important to decide whether funoran can be used as an anti-adhesion agent, biofilm modulator, a constituent of local delivery systems, or a structural constituent in composite anti-caries materials. Research progress can only be achieved by placing funoran in a comprehensive model that includes cariogenic processes, microecological homeostasis, and material translation between initial observation of activity and mechanistic insight and practical engineering.

### 3.1 Limitations and controversies in existing research

Although the potential of funoran and related algal polysaccharides is promising in the prevention of oral caries, the existing evidence base is not yet sufficiently deep or rigorous. In the case of funoran, direct research remains uncommon, and much of the research conducted has been based upon anti-adhesion or short-term plaque-related observations. There is still a lack of systematic evidence on its MIC, MBC, MBIC, MBEC, modulation of virulence factors and mechanisms of action in multispecies biofilms (Inagaki et al., 2011; Nakamura et al., 2022; Wang et al., 2014). Therefore, the existing information about funoran is mainly descriptive and its exact role in the regulation of cariogenic biofilms has not been clearly established yet.

An even more general weakness of this area is the lack of standardization of the research on algal polysaccharides. Differences in algal species, extraction methods, purification, molecular-weight distribution, and degree of sulfation can significantly affect physicochemical properties and biological activity, thereby compromising interstudy comparability (Cheng et al., 2015; Jeon et al., 2011). Further complicating this issue, the analytical design of individual studies, such as the selection of bacterial strains, inoculum conditions, sugar concentrations, biofilm maturation times, and outcome measures, vary, despite the utilization of similar assays, such as MIC, MBC, agar diffusion, biofilm quantification, adhesion assays, microscopic observation, colony-forming unit (CFU) counting, gene expression. These same issues have been highlighted in more general discourse of marine polysaccharide standardization and reproducibility (Cotas et al., 2020; Lomartire and Goncalves, 2022). Consequently, interpretability and generalizability of such results are limited even in cases where antibacterial activity or biofilm reduction has been reported.

The available evidence also remains mechanistically thin. For funoran, the clearest direct evidence still comes from studies showing reduced adhesion of oral streptococci and suppression of *S. mutans* biofilm formation when it is combined with xylitol (Inagaki et al., 2011; Nakamura et al., 2022; Wang et al., 2014). By contrast, its effects on acid production, EPS biosynthesis, glucosyltransferase activity, and virulence-gene expression have not yet been directly characterized. Funoran is still less well characterized than several natural agents that have been studied more extensively in caries-related models. For chitosan, available evidence includes systematic assessment of its effect on salivary *S. mutans* (Róna et al., 2023). Green tea catechins have been reviewed in relation to *S. mutans* growth, biofilm formation, and virulence-associated activity (Aragão et al., 2022; Kong et al., 2022). Cranberry-derived proanthocyanidins have also been assessed for their effects on *S. mutans*-related microbiological activity (Castellanos et al., 2024). By comparison, funoran still lacks standardized effective concentration ranges, systematic toxicity assessment in oral formulations, and robust clinical validation. Future studies should therefore benchmark funoran against these agents using comparable adhesion, EPS, acidogenicity, biofilm, and clinical-readout models.

Evidence from related marine sulfated polysaccharides offers only partial guidance, as these studies suggest that algal polysaccharides can reduce biofilm accumulation, interfere with matrix organization, and influence virulence-associated behavior in oral or biofilm-related models (Achmad et al., 2020; Jun et al., 2018; Lin et al., 2021; Zhang et al., 2021). Such findings are useful for framing hypotheses, but they should not be treated as evidence that funoran acts through the same mechanisms. Future work should therefore examine whether funoran changes lactic-acid production, terminal biofilm pH, water-soluble and water-insoluble EPS levels, and glucosyltransferase activity. Gene-level assays are also needed, particularly for targets involved in adhesion, EPS formation, acid tolerance, and biofilm maturation, including *gtfB*, *gtfC*, *gtfD*, *gbpB*, *spaP*, *ftf*, *brpA*, and *vicR*. These endpoints would clarify whether funoran mainly blocks the earliest adhesion step or whether it also affects downstream cariogenic virulence.

Important translational gaps also remain. Despite the significant potential in a variety of delivery systems, including chewing gum, mouthwash, nanoparticles, hydrogels, and intelligent particles, which have demonstrated significant promise in other natural-product and polysaccharide systems, mature funoran-focused oral products are still lacking, and clinical and *in vivo* evidence remains relatively scarce (Amissah et al., 2021; Ancuceanu et al., 2019). Recent clinical trials of related marine polysaccharides, including fucoidan-based gut microbiome interventions and carrageenan-containing mucosal spray formulations, indicate that sulfated marine polysaccharides can be translated into human-use settings, but these studies remain outside the direct oral-caries context (Garcia et al., 2025; Unger-Manhart et al., 2024; Wang et al., 2024). The further development of material-based oral delivery systems is more recent, but it highlights this translational gap of funoran (Chen et al., 2025; He et al., 2024; Weng et al., 2022). Meanwhile, there remains doubt as to whether future candidates need to be engineered to achieve strong broad-spectrum antibacterial activity or whether they should be engineered to be mild and selective in ecological regulation. If the major benefit of funoran is decreased adhesion, biofilm destabilization, and reduced localized cariogenic risk, then its value may lie not in direct bactericidal efficacy but in longer-term regulatory compatibility with oral microecological defense. However, even in multispecies systems, long-term exposure models, and host-involved experimental systems, this hypothesis still needs to be confirmed before it can offer a dependable mechanistic interpretation and product development basis.

### 3.2 Research prospects

The way forward in funoran research is initially to have in place a more stringent system of structural characterization and quality control. Important parameters such as source algal species, extraction route, purification procedure, molecular weight distribution, degree of sulfation, monosaccharide composition and conformational features must be characterized and reported systematically and in a standardized fashion so as to facilitate future structure–activity relationship studies. It is only under conditions where material comparability can be achieved that one can be assured of reliable interpretation of findings across studies and stronger attribution of differences in activity to inherent structural characteristics and not to differences in the preparation procedures or sample quality. Simultaneously, a standardized system of *in vitro* assessments of the effects of funoran on *S. mutans*, *S. sobrinus*, *A. viscosus*, *L. acidophilus*, and mixed microbial communities in the planktonic condition and biofilm should be established. The key endpoints, such as MIC, MBC, MBIC, MBEC, adhesion, EPS production, acidogenicity, microscopic architecture, and associated gene expression, must be reported as homogenously as possible to maximize the interstudy comparability and integration of evidence. To make these recommendations more operational, Table 3 proposes a standardized evaluation roadmap covering material characterization, early adhesion testing, mature biofilm analysis, EPS and acidogenicity assessment, ecological models, and formulation-oriented translation.

**Table 3:** Standardized evaluation roadmap and recommended reporting items for future funoran studies.

Stage	Recommended models	Priority endpoints	Key reporting items
Material characterization	Purified funoran fractions; batch-to-batch comparison	Molecular weight, degree of sulfation, monosaccharide composition, viscosity, pH, conformation	Algal source, extraction method, purification process, batch number, analytical method
Baseline antibacterial testing	Planktonic *S. mutans*, *S. sobrinus*, *A. viscosus*, *L. acidophilus*	MIC, MBC, growth curve, viable counts	Strain source, inoculum density, medium, exposure time, funoran concentration
Early adhesion model	Saliva-coated hydroxyapatite, enamel, or dentin surfaces	Adhesion rate, CFU, surface coverage, early biomass	Pellicle preparation, substratum type, treatment timing, washing protocol
Mature biofilm model	Static microplate biofilm, 3D biofilm, flow-cell system	MBIC, MBEC, biofilm thickness, biomass, bacterial viability	Biofilm age, sucrose concentration, treatment mode, replicate number
EPS and acidogenicity assessment	*S. mutans* or multispecies cariogenic biofilms	Water-soluble EPS, water-insoluble EPS, glucosyltransferase activity, lactic acid, terminal pH	EPS assay method, pH measurement time, normalization method
Structural and microscopic analysis	confocal laser scanning microscopy (CLSM), scanning electron microscopy (SEM), live/dead staining	Biofilm architecture, live/dead distribution, matrix organization	Imaging settings, quantified fields, image-analysis method
Ecological relevance	Multispecies biofilms, saliva-derived microcosm biofilms, *in situ* enamel-slab models	Microbial composition, acid production, enamel demineralization, commensal compatibility	Species composition, donor saliva source, ethical approval if applicable
Formulation and translation	Chewing gum, mouthwash, oral film, hydrogel, nanoparticle system	Release profile, retention, stability, cytocompatibility, enamel/mucosal safety	Vehicle control, formulation composition, exposure frequency, safety assay

On the level of experimental design, the future research should focus on fundamental scientific questions instead of the further repetitive screening of activities. In cases where it is of interest to assess funoran as an anti-adhesion agent, experimental models ought to be more reminiscent of early oral colonization, including salivary pellicle-coated surfaces and models of early colonization competition, to better capture the overall impact of funoran on early attachment and community formation. To establish the long-term efficacy of antibiofilm formulations, more focus should be on mature biofilms, multispecies communities, and long-term repeated-exposure models when the objective is to develop antibiofilm formulations. In the event that there is a shift in focus toward product development, the parameters of practical application should also be researched, such as stability, local retention, taste acceptability and formulation compatibility. The proposed experimental strategies, then, ought to be in line with the desired situation of application, allowing research on funoran to move beyond the question of whether it is an effective agent to exploring where it is most effective and where it is most suitable.

In addition to *in vitro* research, more focus should also be given to animal research and preclinical validation with special consideration to the long-term outcomes of funoran on caries development, the salivary microenvironment, and the normal oral microbiota. For a natural polysaccharide intended for long-term local application, short-term plaque reduction or prevention of specific cariogenic indicators is not enough. More importantly, there is a need to establish whether funoran can maintain the oral microecological equilibrium in the long term and without any negative consequences on the host environment. Future work should therefore bridge recent multispecies biofilm models with staged *in vivo* and clinical designs, rather than extrapolating directly from single-species or non-oral clinical evidence (Dang et al., 2024; Huang et al., 2025; Pudipeddi et al., 2025; Garcia et al., 2025; Wang et al., 2024). Meanwhile, its development must not be limited to a one-component strategy, but it must also increasingly explore combinations with xylitol, probiotics, nanocarriers, hydrogels, and biomimetic remineralization systems. Integration of complementary functional modules can allow funoran to have synergistic effects on anti-adhesion, biofilm regulation, local delivery, and remineralization and enhance its translational potential.

These steps are necessary to achieve high-quality systematic reviews, meta-analyses, and evidence-based assessment. More broadly, the importance of funoran research is not only the demonstration of another algae-derived caries-preventive candidate, but an improvement of the direction of the field to a more comprehensive strategy that focuses on mechanisms, materials, and ecological control. Its value may lie less in being the most effective antibacterial agent than in serving as an effective bridge between the study of cariogenic mechanisms, development of marine natural products and targeted local oral therapy. This is why, in the future, research must be aimed at identifying its functional positioning, enhancing mechanistic data, and facilitating application-based integration, instead of reiterating its nonspecific bioactivity.

## 4 Conclusion

In summary, caries studies in the last 15 years have increasingly moved beyond a one-cariogenic-bacterium paradigm to a multifactorial model of microecological dysbiosis, biofilm development, and networks of virulence. In this regard, funoran, a red algae-derived sulfated polysaccharide, has been proposed as a promising natural option for caries prevention due to its anti-adhesive properties, possible anti-biofilm properties, and good material interactions. There is some direct evidence available that funoran is capable of preventing the adhesion of oral streptococci and that it can have more biofilm-inhibitory activity when used with xylitol. Parallel investigations of related algal polysaccharides also enhance the overall prospects of marine polysaccharides in antibacterial and biofilm control, anti-inflammatory activity, and oral-delivery applications.

There is still limited direct experimental evidence, and its structure–activity relationship has not been well defined, methodology standards are still inconsistent, and there is insufficient support for clinical translation. In this regard, funoran is currently better considered as a novel algal polysaccharide candidate with evident research potential that needs additional validation with high-quality evidence. If future research can create a comprehensive research framework that includes structural characterization, standardized *in vitro* testing, mechanistic analysis, animal testing, and oral delivery, funoran can be translated into a new anti-caries intervention tool that will combine biological and material benefits.

## References

[ref1] Achmad H. Huldani Ramadhany Y. F. (2020). Antimicrobial activity and sulfated polysaccharides antibiofilms in marine algae against dental plaque bacteria: a literature review. Syst. Rev. Pharm. 11, 459–465. doi: 10.31838/srp.2020.6.72

[ref2] AlEraky D. M. Madi M. El Tantawi M. AlHumaid J. Fita S. AbdulAzeez S. . (2021). Predominance of non-*Streptococcus mutans* bacteria in dental biofilm and its relation to caries progression. Saudi J. Biol. Sci. 28, 7390–7395. doi: 10.1016/j.sjbs.2021.08.052, 34867042 PMC8626303

[ref3] Amissah F. Andey T. Ahlschwede K. M. (2021). Nanotechnology-based therapies for the prevention and treatment of *Streptococcus mutans*-derived dental caries. J. Oral. Biosci. 63, 327–336. doi: 10.1016/j.job.2021.09.002, 34536629

[ref4] Ancuceanu R. Anghel A. I. Ionescu C. Hovanet M. V. Cojocaru-Toma M. Dinu M. (2019). Clinical trials with herbal products for the prevention of dental caries and their quality: a scoping study. Biomolecules. 9:884. doi: 10.3390/biom9120884, 31861065 PMC6995540

[ref5] Aragão M. G. B. Aires C. P. Corona S. A. M. (2022). Effects of the green tea catechin epigallocatechin-3-gallate on *Streptococcus mutans* planktonic cultures and biofilms: systematic literature review of in vitro studies. Biofouling 38, 687–695. doi: 10.1080/08927014.2022.2116320, 36017657

[ref6] Azarm A. Ayoobi F. Zare-Bidaki M. Taheri M. Zarandi E. R. (2024). Antibacterial and antibiofilm activities of *Tribulus terrestris* methanolic extract against *Streptococcus mutans*, Streptococcus sobrinus, and *Lactobacillus acidophilus*: an in vitro study. Dent. Res. J. 21:57. doi: 10.4103/drj.drj_518_23, 39574561 PMC11581358

[ref7] Aziz E. Batool R. Khan M. U. Rauf A. Akhtar W. Heydari M. . (2021). An overview on red algae bioactive compounds and their pharmaceutical applications. J. Complement. Integr. Med. 17:20190203. doi: 10.1515/jcim-2019-0203, 32697756

[ref8] Banas J. A. Drake D. R. (2018). Are the mutans streptococci still considered relevant to understanding the microbial etiology of dental caries? BMC Oral Health 18:129. doi: 10.1186/s12903-018-0595-2, 30064426 PMC6069834

[ref9] Behzadnia A. Moosavi-Nasab M. Oliyaei N. (2024). Anti-biofilm activity of marine algae-derived bioactive compounds. Front. Microbiol. 15:1270174. doi: 10.3389/fmicb.2024.1270174, 38680918 PMC11055458

[ref10] Bowen W. Koo H. (2011). Biology of *Streptococcus mutans*-derived glucosyltransferases: role in extracellular matrix formation of cariogenic biofilms. Caries Res. 45, 69–86. doi: 10.1159/000324598, 21346355 PMC3068567

[ref11] Brady A. J. Laverty G. Gilpin D. F. Kearney P. Tunney M. (2017). Antibiotic susceptibility of planktonic- and biofilm-grown staphylococci isolated from implant-associated infections: should MBEC and nature of biofilm formation replace MIC? J. Med. Microbiol. 66, 461–469. doi: 10.1099/jmm.0.000466, 28463662

[ref12] Castellanos J. S. Betancourt D. E. Díaz-Báez D. Baldión P. A. (2024). Effect of flavonoids from grape seed and cranberry extracts on the microbiological activity of *Streptococcus mutans*: a systematic review of in vitro studies. BMC Oral Health 24:662. doi: 10.1186/s12903-024-04263-0, 38840232 PMC11155149

[ref13] Chen X. Daliri E. B. M. Kim N. Kim J. R. Yoo D. Oh D. H. (2020). Microbial etiology and prevention of dental caries: exploiting natural products to inhibit cariogenic biofilms. Pathogens. 9:569. doi: 10.3390/pathogens9070569, 32674310 PMC7400585

[ref14] Chen Y. Lin S. Huang X. Zhou W. (2025). From biofilm control to biomimetic remineralization: hydrogels in prevention and treatment of dental caries. Front. Cell. Infect. Microbiol. 15:1663563. doi: 10.3389/fcimb.2025.1663563, 41040988 PMC12484240

[ref15] Chen L. Ren Z. Zhou X. Zeng J. Zou J. Li Y. (2016). Inhibition of *Streptococcus mutans* biofilm formation, extracellular polysaccharide production, and virulence by an oxazole derivative. Appl. Microbiol. Biot. 100, 857–867. doi: 10.1007/s00253-015-7092-1, 26526453

[ref16] Cheng L. Li J. He L. Zhou X. (2015). Natural products and caries prevention. Caries Res. 49, 38–45. doi: 10.1159/000377734, 25871417

[ref17] Cotas J. Leandro A. Pacheco D. Goncalves A. M. M. Pereira L. (2020). A comprehensive review of the nutraceutical and therapeutic applications of red seaweeds (Rhodophyta). Life. 10:19. doi: 10.3390/life10030019, 32110890 PMC7151636

[ref18] Dang M.-H. Cai J.-N. Choi H.-M. Kim D. Oh H.-W. Jeon J.-G. (2024). Difference in formation of a dental multi-species biofilm according to substratum direction. Arch. Oral Biol. 164:106002. doi: 10.1016/j.archoralbio.2024.106002, 38759390

[ref19] Fragkou S. Balasouli C. Tsuzukibashi O. Argyropoulou A. Menexes G. Kotsanos N. . (2016). *Streptococcus mutans*, Streptococcus sobrinus and *Candida albicans* in oral samples from caries-free and caries-active children. Eur. Arch. Paediatr. Dent. 17, 367–375. doi: 10.1007/s40368-016-0239-7, 27357362

[ref20] Galvao L. C. D. C. Furletti V. F. Bersan S. M. F. da Cunha M. G. Ruiz A. L. T. G. Carvalho J. E. D. . (2012). Antimicrobial activity of essential oils against Streptococcus mutans and their antiproliferative effects. Evid. Based Complement. Alternat. Med. 2012, 1–12. doi: 10.1155/2012/751435, 22685486 PMC3368214

[ref21] Gao L. Liu Y. Kim D. Li Y. Hwang G. Naha P. C. . (2016). Nanocatalysts promote *Streptococcus mutans* biofilm matrix degradation and enhance bacterial killing to suppress dental caries in vivo. Biomaterials 101, 272–284. doi: 10.1016/j.biomaterials.2016.05.051, 27294544 PMC4949957

[ref22] Garcia G. Soto J. Valenzuela C. Bernal M. Barreto J. Luzardo M. . (2025). Gut microbiome modulation and health benefits of a novel fucoidan extract from Saccharina latissima: a double-blind, placebo-controlled trial. Microorganisms 13:1545. doi: 10.3390/microorganisms13071545, 40732054 PMC12298129

[ref23] Golus J. Sawicki R. Widelski J. Ginalska G. (2016). The agar microdilution method-a new method for antimicrobial susceptibility testing for essential oils and plant extracts. J. Appl. Microbiol. 121, 1291–1299. doi: 10.1111/jam.13253, 27501239

[ref24] Hallmann L. Gerngroß M. D. (2025). Antibacterial polysaccharides in dental implantology. Mar. Drugs 23:321. doi: 10.3390/md23080321, 40863638 PMC12388009

[ref25] He Y. Bright R. Vasilev K. Zilm P. (2024). Development of “intelligent particles” for the treatment of dental caries. Eur. J. Pharm. Biopharm. 202:114374. doi: 10.1016/j.ejpb.2024.114374, 38942176

[ref26] He Z. Huang Z. Jiang W. Zhou W. (2019). Antimicrobial activity of cinnamaldehyde on *Streptococcus mutans* biofilms. Front. Microbiol. 10:2241. doi: 10.3389/fmicb.2019.02241, 31608045 PMC6773874

[ref27] Hoceini A. Klouche Khelil N. Ben-Yelles I. Mesli A. Ziouani S. Ghellai L. . (2016). Caries-related factors and bacterial composition of supragingival plaques in caries-free and caries-active Algerian adults. Asian Pac. J. Trop. Biomed. 6, 720–726. doi: 10.1016/j.apjtb.2016.06.011

[ref28] Huang Y. Z. Jin Z. Wang Z. M. Qi L. B. Song S. Zhu B. W. . (2021). Marine bioactive compounds as nutraceutical and functional food ingredients for potential oral health. Front. Nutr. 8:686663. doi: 10.3389/fnut.2021.686663, 34926539 PMC8675007

[ref29] Huang F. Zhou Y. Chen D. Lin H. (2025). Anti-cariogenic activity of mutanocyclin, a secondary metabolite of *Streptococcus mutans*, in mono- and multispecies biofilms. Microbiol. Spectrum 13, e00183–e00125. doi: 10.1128/spectrum.00183-25, 40558055 PMC12323323

[ref30] Inagaki S. Saeki Y. Ishihara K. (2011). Funoran-containing xylitol gum and tablets inhibit adherence of oral streptococci. J. Oral. Biosci. 53, 82–86. doi: 10.1016/S1349-0079(11)80039-3

[ref31] Jeon J. G. Rosalen P. Falsetta M. Koo H. (2011). Natural products in caries research: current (limited) knowledge, challenges and future perspective. Caries Res. 45, 243–263. doi: 10.1159/000327250, 21576957 PMC3104868

[ref32] Joycharat N. Limsuwan S. Subhadhirasakul S. Voravuthikunchai S. P. Pratumwan S. Madahin I. . (2012). Anti-*Streptococcus mutans* efficacy of Thai herbal formula used as a remedy for dental caries. Pharm. Biol. 50, 941–947. doi: 10.3109/13880209.2011.649430, 22489572

[ref33] Jun J. Y. Jung M. J. Jeong I. H. Yamazaki K. Kawai Y. Kim B. M. (2018). Antimicrobial and antibiofilm activities of sulfated polysaccharides from marine algae against dental plaque bacteria. Mar. Drugs 16:301. doi: 10.3390/md16090301, 30150576 PMC6165115

[ref34] Kanmani P. Rhim J. W. (2014). Antimicrobial and physical-mechanical properties of agar-based films incorporated with grapefruit seed extract. Carbohydr. Polym. 102, 708–716. doi: 10.1016/j.carbpol.2013.10.099, 24507339

[ref35] Katsarov P. Shindova M. Lukova P. Belcheva A. Delattre C. Pilicheva B. (2021). Polysaccharide-based micro- and nanosized drug delivery systems for potential application in the pediatric dentistry. Polymers 13:3342. doi: 10.3390/polym13193342, 34641160 PMC8512615

[ref36] Kim Y. H. Bang Y. J. Yoon K. S. Priyadarshi R. Rhim J. W. (2022). Pine needle (*Pinus densiflora*) extract-mediated synthesis of silver nanoparticles and the preparation of carrageenan-based antimicrobial packaging films. J. Nanomater. 2022:8395302. doi: 10.1155/2022/8395302

[ref37] Kong C. Zhang H. Li L. Liu Z. (2022). Effects of green tea extract epigallocatechin-3-gallate (EGCG) on oral disease-associated microbes: a review. J. Oral Microbiol. 14:2131117. doi: 10.1080/20002297.2022.2131117, 36212989 PMC9542882

[ref38] Koo H. Bowen W. H. (2014). Candida albicans and *Streptococcus mutans*: a potential synergistic alliance to cause virulent tooth decay in children. Future Microbiol. 9, 1295–1297. doi: 10.2217/fmb.14.92, 25517895

[ref39] Korona-Glowniak I. Skawinska-Bednarczyk A. Wrobel R. Pietrak J. Tkacz-Ciebiera I. Maslanko-Switala M. . (2022). *Streptococcus sobrinus* as a predominant oral bacteria related to the occurrence of dental caries in polish children at 12 years old. Int. J. Environ. Res. Public Health 19:15005. doi: 10.3390/ijerph192215005, 36429724 PMC9690266

[ref40] Krzysciac W. Jurczak A. Koscielniak D. Bystrowska B. Skalniak A. (2014). The virulence of Streptococcus mutans and the ability to form biofilms. Eur. J. Clin. Microbiol. Infect. Dis. 33, 499–515. doi: 10.1007/s10096-013-1993-7, 24154653 PMC3953549

[ref41] Lee S. H. Kim Y. J. (2014). A comparative study of the effect of probiotics on cariogenic biofilm model for preventing dental caries. Arch. Microbiol. 196, 601–609. doi: 10.1007/s00203-014-0998-7, 24919536

[ref42] Lemos J. Palmer S. Zeng L. Wen Z. Kajfasz J. Freires I. . (2019). The biology of *Streptococcus mutans*. Microbiol. Spectrum 7:GPP3-0051-2018. doi: 10.1128/microbiolspec.GPP3-0051-2018, 30657107 PMC6615571

[ref43] Liang N. L. Luo B. W. Sun I. G. Chu C. H. Duangthip D. (2024). Clinical effects of sugar substitutes on cariogenic bacteria: a systematic review and meta-analysis. Int. Dent. J. 74, 987–998. doi: 10.1016/j.identj.2024.02.008, 38599933 PMC11561516

[ref44] Lin Y. Chen J. Zhou X. Li Y. (2021). Inhibition of *Streptococcus mutans* biofilm formation by strategies targeting the metabolism of exopolysaccharides. Crit. Rev. Microbiol. 47, 667–677. doi: 10.1080/1040841X.2021.1915959, 33938347

[ref45] Lin G. S. S. Cher C. Y. Goh Y. H. Chan D. Z. K. Karobari M. I. Lai J. C. H. . (2022). An insight into the role of marine biopolymer alginate in endodontics: a review. Mar. Drugs 20:539. doi: 10.3390/md20080539, 36005542 PMC9409890

[ref46] Liu Z. Gao T. Yang Y. Meng F. Zhan F. Jiang Q. . (2019). Anti-cancer activity of porphyran and carrageenan from red seaweeds. Molecules 24:4286. doi: 10.3390/molecules24234286, 31775255 PMC6930528

[ref47] Lomartire S. Goncalves A. M. M. (2022). An overview of potential seaweed-derived bioactive compounds for pharmaceutical applications. Mar. Drugs 20:141. doi: 10.3390/md20020141, 35200670 PMC8875101

[ref48] Lu Y. Lin Y. Li M. He J. (2023). Roles of *Streptococcus mutans*-*Candida albicans* interaction in early childhood caries: a literature review. Front. Cell. Infect. Microbiol. 13:1151532. doi: 10.3389/fcimb.2023.1151532, 37260705 PMC10229052

[ref49] Madruga L. Y. Sabino R. M. Santos E. C. Popat K. C. Balaban R. D. C. Kipper M. J. (2020). Carboxymethyl-kappa-carrageenan: a study of biocompatibility, antioxidant and antibacterial activities. Int. J. Biol. Macromol. 152, 483–491. doi: 10.1016/j.ijbiomac.2020.02.274 32109473

[ref50] Matias Regis W. F. Ruliglesio Rocha F. Araujo Lima R. Panariello B. Duarte S. da Cunha Costa A. . (2025). Insights into the role of Streptococcus mutans and *Candida albicans* in dental biofilm formation and cariogenicity: a literature review. Cureus. 17:e86159. doi: 10.7759/cureus.86159, 40672011 PMC12266937

[ref51] Matin M. Koszarska M. Atanasov A. G. Krol-Szmajda K. Jozwik A. Stelmasiak A. . (2024). Bioactive potential of algae and algae-derived compounds: focus on anti-inflammatory, antimicrobial, and antioxidant effects. Molecules 29:4695. doi: 10.3390/molecules29194695, 39407623 PMC11477577

[ref52] Matsumoto-Nakano M. (2018). Role of *Streptococcus mutans* surface proteins for biofilm formation. Jpn. Dent. Sci. Rev. 54, 22–29. doi: 10.1016/j.jdsr.2017.08.002, 29628998 PMC5884221

[ref53] Mattos-Graner R. O. Klein M. I. Smith D. J. (2014). Lessons learned from clinical studies: roles of mutans streptococci in the pathogenesis of dental caries. Curr. Oral Health Rep. 1, 70–78. doi: 10.1007/s40496-013-0008-1

[ref54] Mazurel D. Brandt B. W. Boomsma M. Crielaard W. Lagerweij M. Exterkate R. A. M. . (2025). Streptococcus mutans and caries: a systematic review and meta-analysis. J. Dent. Res. 104, 594–603. doi: 10.1177/00220345241303880, 39895020 PMC12075887

[ref55] Metwalli K. H. Khan S. A. Krom B. P. Jabra-Rizk M. A. (2013). *Streptococcus mutans*, Candida albicans, and the human mouth: a sticky situation. PLoS Pathog. 9:e1003616. doi: 10.1371/journal.ppat.1003616, 24146611 PMC3798555

[ref56] Momeni S. S. Cao X. Xie B. Rainey K. Childers N. K. Wu H. (2024). Intraspecies interactions of *Streptococcus mutans* impact biofilm architecture and virulence determinants in childhood dental caries. mSphere 9:e00778-23, e00778–e00723. doi: 10.1128/msphere.00778-23, 38990043 PMC11288028

[ref57] Motallaei M. N. Yazdanian M. Tebyanian H. Tahmasebi E. Alam M. Abbasi K. . (2021). The current strategies in controlling oral diseases by herbal and chemical materials. Evid. Based Complement. Alternat. Med. 2021, 1–22. doi: 10.1155/2021/3423001, 34471415 PMC8405301

[ref58] Nagarathna C. Veena R. (2020). Correlation of *Streptococcus mutans* and *Streptococcus sobrinus* colonization with and without caries experience in preschool children. Indian J. Dent. Res. 31, 73–79. doi: 10.4103/ijdr.IJDR_432_18, 32246686

[ref59] Najafi S. Mardani M. Motamedifar M. Nazarinia M. A. Hadadi M. (2022). Salivary Streptococcus mutans and lactobacilli levels as indicators of dental caries development in iranian patients with systemic sclerosis. Iran. J. Med. Microbiol. 16, 350–356. doi: 10.30699/ijmm.16.4.350

[ref60] Nakamura T. Yonezawa H. Kawarai T. Narisawa N. Senpuku H. (2022). Inhibitory effect of the combination of xylitol and funoran on *Streptococcus mutans* biofilm formation on the uncoated surface. Arch. Microbiol. 204:723. doi: 10.1007/s00203-022-03299-6, 36416971

[ref61] Niu Y. Wang K. Zheng S. Wang Y. Ren Q. Li H. . (2020). Antibacterial effect of caffeic acid phenethyl ester on cariogenic bacteria and *Streptococcus mutans* biofilms. Antimicrob. Agents Chemother. 64, e00251–e00220. doi: 10.1128/AAC.00251-20, 32540977 PMC7449213

[ref62] Palafox Felix S. Sandoval Larios G. Cabrera R. Garcia-Galaz A. Huerta-Ocampo J. A. Guzman-Partida A. M. . (2025). Effects of fucoidan and fucoidan oligosaccharides in growth and quorum sensing mediated virulence factor of *Campylobacter jejuni*. Polysaccharides 6:24. doi: 10.3390/polysaccharides6020024

[ref63] Pei Y. Yang S. Xiao Z. Zhou C. Hong P. Qian Z. J. (2021). Structural characterization of sulfated polysaccharide isolated from red algae (*Gelidium crinale*) and antioxidant and anti-inflammatory effects in macrophage cells. Front. Bioeng. Biotechnol. 9:794818. doi: 10.3389/fbioe.2021.794818, 34869300 PMC8637441

[ref64] Perez M. Falque E. Dominguez H. (2016). Antimicrobial action of compounds from marine seaweed. Mar. Drugs 14:52. doi: 10.3390/md14030052, 27005637 PMC4820306

[ref65] Perez-Diaz M. A. Boegli L. James G. Velasquillo C. Sanchez-Sanchez R. Martinez-Martinez R. E. . (2015). Silver nanoparticles with antimicrobial activities against Streptococcus mutans and their cytotoxic effect. Mat. Sci. Eng. C. 55, 360–366. doi: 10.1016/j.msec.2015.05.036, 26117766

[ref66] Pudipeddi A. Bijle M. N. Yiu C. (2025). Effect of arginine-based synbiotics on multispecies biofilm. J. Dent. 161:105974. doi: 10.1016/j.jdent.2025.105974 40653001

[ref67] Qiu Y. Jiang H. Fu L. Ci F. Mao X. (2021). Porphyran and oligo-porphyran originating from red algae Porphyra: preparation, biological activities, and potential applications. Food Chem. 349:129209. doi: 10.1016/j.foodchem.2021.129209, 33588184

[ref68] Róna V. Bencze B. Kelemen K. Végh D. Tóth R. Kói T. . (2023). Effect of chitosan on the number of *Streptococcus mutans* in saliva: a meta-analysis and systematic review. Int. J. Mol. Sci. 24:15270. doi: 10.3390/ijms242015270, 37894948 PMC10607225

[ref69] Rudin L. Bornstein M. M. Shyp V. (2023). Inhibition of biofilm formation and virulence factors of cariogenic oral pathogen *Streptococcus mutans* by natural flavonoid phloretin. J. Oral Microbiol. 15:2230711. doi: 10.1080/20002297.2023.2230711, 37416858 PMC10321187

[ref70] Sangavi R. Malligarjunan N. Satish L. Raja V. Pandian S. K. Gowrishankar S. (2024). Anticariogenic activity of marine brown algae Padina boergesenii and its active components towards *Streptococcus mutans*. Front. Cell. Infect. Microbiol. 14:1458825. doi: 10.3389/fcimb.2024.1458825, 39654980 PMC11625749

[ref71] Schumacher A. Vranken T. Malhotra A. Arts J. J. C. Habibovic P. (2018). *In vitro* antimicrobial susceptibility testing methods: agar dilution to 3D tissue-engineered models. Eur. J. Clin. Microbiol. 37, 187–208. doi: 10.1007/s10096-017-3089-2, 28871407 PMC5780537

[ref72] Shankar S. Reddy J. P. Rhim J. W. Kim H. Y. (2015). Preparation, characterization, and antimicrobial activity of chitin nanofibrils reinforced carrageenan nanocomposite films. Carbohydr. Polym. 117, 468–475. doi: 10.1016/j.carbpol.2014.10.010, 25498660

[ref73] Simona J. Dani D. Petr S. Marcela N. Jakub T. Bohuslava T. (2021). Edible films from carrageenan/orange essential oil/trehalose-structure, optical properties, and antimicrobial activity. Polymers 13:332. doi: 10.3390/polym13030332, 33494246 PMC7864528

[ref74] Simon-Soro A. Mira A. (2015). Solving the etiology of dental caries. Trends Microbiol. 23, 76–82. doi: 10.1016/j.tim.2014.10.010, 25435135

[ref75] Song Y. M. Zhou H. Y. Wu Y. Wang J. Liu Q. Mei Y. F. (2020). In vitro evaluation of the antibacterial properties of tea tree oil on planktonic and biofilm-forming *Streptococcus mutans*. AAPS PharmSciTech 21:227. doi: 10.1208/s12249-020-01753-6, 32767025

[ref76] Spatafora G. Li Y. He X. Cowan A. Tanner A. C. R. (2024). The evolving microbiome of dental caries. Microorganisms. 12:121. doi: 10.3390/microorganisms12010121, 38257948 PMC10819217

[ref77] Tabassum N. Khan F. Kang M. G. Jo D. M. Cho K. J. Kim Y. M. (2023). Inhibition of polymicrobial biofilms of *Candida albicans*-*Staphylococcus aureus*/*Streptococcus mutans* by fucoidan-gold nanoparticles. Mar. Drugs 21:123. doi: 10.3390/md21020123, 36827164 PMC9965608

[ref78] Unger-Manhart N. Morokutti-Kurz M. Zieglmayer P. Lemell P. Savli M. Zieglmayer R. (2024). Carrageenan-containing nasal spray alleviates allergic symptoms in participants with grass pollen allergy: a randomized, controlled, crossover clinical trial. Int. J. Gen. Med. 17, 419–428. doi: 10.2147/IJGM.S447359, 38333020 PMC10850985

[ref79] Wang M. J. Chang W. J. Lee W. F. Lee S. Y. Sheu J. R. Teng N. C. (2014). The effects of funoran-containing xylitol chewing gum on dental plaque. J. Polym. Eng. 34, 203–208. doi: 10.1515/polyeng-2013-0225

[ref80] Wang Y. Luo J. Xu C. Hu D. Li Y. Ye Y. . (2026). Recent advance in marine polysaccharides: structure, anti-inflammatory mechanisms, and functional applications. Mar. Drugs 24:129. doi: 10.3390/md24040129, 42042203 PMC13117732

[ref81] Wang H. Wei W. Liu F. Wang M. Zhang Y. Du S. . (2024). Effects of fucoidan and synbiotics supplementation during bismuth quadruple therapy of *Helicobacter pylori* infection on gut microbial homeostasis: an open-label, randomized clinical trial. Front. Nutr. 11:1407736. doi: 10.3389/fnut.2024.1407736, 39010853 PMC11246856

[ref82] Wasfi R. Abd El-Rahman O. A. Zafer M. M. Ashour H. M. (2018). Probiotic *Lactobacillus* sp. inhibit growth, biofilm formation and gene expression of caries-inducing *Streptococcus mutans*. J. Cell. Mol. Med. 22, 1972–1983. doi: 10.1111/jcmm.13496, 29316223 PMC5824418

[ref83] Wei Y. Zhang Y. Zhuang Y. Tang Y. Nie H. Huang Y. . (2024). *Veillonella parvula* acts as a pathobiont promoting the biofilm virulence and cariogenicity of *Streptococcus mutans* in adult severe caries. Microbiol. Spectrum 12, e04318–e04323. doi: 10.1128/spectrum.04318-23, 39345197 PMC11537095

[ref84] Weng L. Wu L. Guo R. Ye J. Liang W. Wu W. . (2022). Lactobacillus cell envelope-coated nanoparticles for antibiotic delivery against cariogenic biofilm and dental caries. J. Nanobiotechnol. 20:356. doi: 10.1186/s12951-022-01563-x, 35918726 PMC9344742

[ref85] Wessel S. W. van der Mei H. C. Maitra A. Dodds M. W. J. Busscher H. J. (2016). Potential benefits of chewing gum for the delivery of oral therapeutics and its possible role in oral healthcare. Expert. Opin. Drug. Del. 13, 1421–1431. doi: 10.1080/17425247.2016.1193154, 27223231

[ref86] Yang Z. Liu W. Liu H. Li R. Chang L. Kan S. . (2022). The applications of polysaccharides in dentistry. Front. Bioeng. Biotechnol. 10:970041. doi: 10.3389/fbioe.2022.970041, 35935501 PMC9355030

[ref87] Yi Y. Wang L. Chen L. Lin Y. Luo Z. Chen Z. . (2020). Farnesal-loaded pH-sensitive polymeric micelles provided effective prevention and treatment on dental caries. J. Nanobiotechnol. 18:89. doi: 10.1186/s12951-020-00633-2, 32527262 PMC7291565

[ref88] Zeng L. Abranches J. Lemos J. A. (2026). A *Streptococcus mutans* retrospective: from oral pathogen to bacterial paradigm. J. Bacteriol. 208, e0055525–e0055525. doi: 10.1128/jb.00555-25, 41685966 PMC13001266

[ref89] Zhang Y. Fang J. Yang J. Gao X. Dong L. Zheng X. . (2022). *Streptococcus mutans*-associated bacteria in dental plaque of severe early childhood caries. J. Oral Microbiol. 14:2046309. doi: 10.1080/20002297.2022.2046309, 35251525 PMC8896182

[ref90] Zhang Q. Ma Q. Wang Y. Wu H. Zou J. (2021). Molecular mechanisms of inhibiting glucosyltransferases for biofilm formation in *Streptococcus mutans*. Int. J. Oral Sci. 13:30. doi: 10.1038/s41368-021-00137-1, 34588414 PMC8481554

[ref91] Zhang Q. Qin S. Xu X. Zhao J. Zhang H. Liu Z. . (2020). Inhibitory effect of *Lactobacillus plantarum* CCFM8724 towards *Streptococcus mutans*- and *Candida albicans*-induced caries in rats. Oxidative Med. Cell. Longev. 2020, 1–10. doi: 10.1155/2020/4345804, 33414892 PMC7769668

[ref92] Zhang Y. Xu Y. Feng X. Xi C. (2025). Relationship between Lactobacillus, *Streptococcus mutans* and caries in early childhood. Int. Dent. J. 75:104444. doi: 10.1016/j.identj.2025.104444

